# Minimal residual disease in systemic light chain amyloidosis: a systematic review and meta-analysis

**DOI:** 10.1007/s00432-024-05733-2

**Published:** 2024-04-15

**Authors:** Xuefeng Li, Yan Yu, Hongbin Yu, Mengran Chen, Xin Zhang, Yu Wu

**Affiliations:** Department of Hematology and Institute of Hematology, West China Hospital, Sichuan University, Chengdu, Sichuan China

**Keywords:** AL amyloidosis, Minimal residual disease, Overall survival, Progression-free survival, Systemic light chain amyloidosis

## Abstract

**Purpose:**

Minimal residual disease (MRD) is a validated prognostic factor in several hematological malignancies. However, its role in systemic light chain (AL) amyloidosis remains controversial, and this systematic review and meta-analysis aims to fill this gap.

**Methods:**

We searched for relevant studies on Pubmed, Embase, and Cochrane Controlled Register of Trials, nine studies involving 451 patients were included and meta-analyzed. This systematic review has been registered in PROSPERO (CRD42023494169).

**Results:**

Our study found that in the group of patients who achieved very good partial response (VGPR) or better, MRD negativity was correlated with higher cardiac and renal response rates [pooled risk ratio (RR) = 0.74 (95% CI 0.62–0.89), 0.74 (95% CI 0.64–0.87), respectively]. Patients with MRD positivity had a higher hematologic progression rate within two years after MRD detection [pooled RR = 10.31 (95% CI 2.02–52.68)]; and a higher risk of hematologic + organ progression in the first year [pooled RR = 12.57 (95% CI 1.73–91.04)]. Moreover, MRD negativity was correlated with a better progression-free survival (PFS) [pooled hazard ratio (HR) = 0.27 (95% CI 0.17–0.45)]; but it did not significantly improve the overall survival (OS) [pooled HR = 0.34 (95% CI 0.11–1.07)].

**Conclusion:**

In AL amyloidosis, our study supports that MRD negativity correlates with higher cardiac or renal response rates and indicates a better PFS in the follow-up. However, the correlation between OS and the status of MRD is not significant.

**Supplementary Information:**

The online version contains supplementary material available at 10.1007/s00432-024-05733-2.

## Introduction

Systemic light chain (AL) amyloidosis is characterized by the secretion of monoclonal immunoglobulin light chains by abnormal clonal plasma cells, which are deposited in target organs, causing organ morphological abnormalities and dysfunction (Gertz 2022; Merlini et al. 2018). The prognosis of AL amyloidosis depends on several factors, including the achievement of hematologic and organ response (Gertz and Dispenzieri 2020; Gertz 2022). Appropriate methods of monitoring the disease during the follow-up can guide the treatment plans, and help to improve patients’ survival or the quality of life.

Minimal residual disease (MRD) has become an important method of monitoring various hematological diseases, and has shown to have important clinical predictive value in some plasma cell disorders, such as multiple myeloma (Medina et al. 2020; Munshi et al. 2020; Perrot et al. 2018). Although AL amyloidosis has a lower clonal plasma cell burden compared to multiple myeloma, a small amount of abnormal free light chains can still be gradually deposited in organs and cause damage (Kastritis et al. 2021b; Saito et al. 2021). Therefore, monitoring the MRD status in AL amyloidosis may be valuable during the treatment and the follow-up. Several studies have reported the relationship between MRD status and organ response rate, progression-free survival (PFS), and overall survival (OS) in AL amyloidosis, but the results were inconsistent. Considering the characteristics of AL deposition, it is unclear whether the MRD negativity can provide a long-term benefit, under the condition of reaching a certain depth of remission. This systematic review aims to conduct a meta-analysis of the currently available data, and provides a higher level of clinical evidence to guide the treatment and monitoring of AL amyloidosis.

## Materials and methods

In this study, we followed the standards set by The Preferred Reporting Items for Systematic Reviews and Meta-Analyses (PRISMA) (Moher et al. 2009). This systematic review has been registered in PROSPERO (CRD42023494169).

### Eligibility criteria

We planned to include studies that evaluated the impact of MRD status on the clinical response to treatment or outcomes during follow-up in AL amyloidosis. Studies that evaluated other diseases (e.g., multiple myeloma) were excluded.

### Literature search

We searched PUBMED, EMBASE, and Cochrane Controlled Register of Trials (CENTRAL) from the study inception to June 3, 2023. We combined Medical Subject Headings terms and free-text terms to search for potential target studies (Supplementary Text S1). Moreover, we reviewed the reference lists of the included studies to identify additional studies.

### Article quality assessment

The methodological quality of each study was assessed via the methodological index for non-randomized studies (MINORS) guidelines (Zeng et al. 2015). MINORS has 12 items, of which 8 apply to both non-comparative and comparative studies, whereas the remaining 4 are exclusively applied to comparative studies. The items applicable for comparative studies include: study aims, consecutive patient inclusion criteria, prospective pooling of data, endpoint consistent with the study aim, unbiased evaluation of endpoints, follow-up period, loss to follow-up less than 5%, prospective calculation of the sample size, an adequate control group, contemporary groups, baseline equivalence of groups and adequate statistical analyses. The items were scored 0 (not reported), 1 (reported but inadequate), or 2 (reported and adequate), and the total score represented the summary assessment of bias risk for each study.

### Definition and MRD detection method

The diagnosis and response criteria of AL amyloidosis were based on “the definition of organ involvement and treatment response in immunoglobulin light chain amyloidosis (AL): A consensus opinion from the 10th International Symposium on Amyloid and Amyloidosis” and “New Criteria for Response to Treatment in Immunoglobulin Light Chain Amyloidosis Based on Free Light Chain Measurement and Cardiac Biomarkers: Impact on Survival Outcomes” (Gertz et al. 2005; Palladini et al. 2012). The risk stratification was based on the Mayo 2004 and/or 2012 system (Dispenzieri et al. 2004; Kumar et al. 2012). The methods of detecting MRD varied in the included studies, which included multi-parametric flow cytometry (MFC) in eight studies, and next generation sequencing (NGS) in one study. The sensitivity of MFC ranged from 2 × 10^–6^ to 1 × 10^–4^; while the sensitivity of NGS was 1 × 10^–6^.

### Statistical analysis

In this study, we used the Meta module in the R-4.3.2 statistical software package to analyze the data. The risk ratio (RR) in each study was calculated according to the number of patients with MRD negativity or positivity. The methodology of calculating the hazard ratio (HR) was based on the recommended method for incorporating summary time-to-event data into meta-analysis shared by Tierney et al. (2007). The p-value of PFS or OS curve, the number of progression or death events, and the number of patients with MRD positivity or negativity reported in the included studies were used to calculate the O-E and Variance, and then the table provided by Tierney et al. (2007) was used to calculate the hazard ratio (Supplementary Text S2). The pooled RR and HR were calculated using the corresponding codes in Meta module.

Heterogeneity was calculated by the I-squared test (I^2^ test). The results were based on the random effects model when heterogeneity was present (I^2^ > 50%); otherwise, the common effect model was chosen. When the upper and lower values of 95% confidence interval (CI) were both less or greater than 1.00, then the pooled RR or HR were considered statistically significant.

## Results

### Literature search results and basic information of included studies

The process of literature search is presented in Supplementary Fig. S1. After excluding duplicates and those that did not meet the inclusion criteria, we ultimately included 9 studies involving 451 patients to perform our meta-analysis (Chakraborty et al. 2022; Diaz-Pallares et al. 2020; Kastritis et al. 2021a; Li et al. 2022; Muchtar et al. 2020; Palladini et al. 2021; Sarosiek et al. 2021; Sidana et al. 2020; Staron et al. 2020). The characteristics of the included studies are illustrated in Table 1. Of the 9 included studies, 8 were retrospective and 1 was prospective. The characteristics of the patients in each included study are illustrated in Supplementary Table 2. Table 2 shows the outcomes of the included studies.

**Table 1:** Characteristics of the included studies

References	Design	Country	Study time period	No. of total patients	No. of VGPR/CR patients at MRD assessment (%)	No. of MRD negative patients (%)	MRD methodology	Sensitivity of MRD assay
Chakraborty et al. (2022)	Retrospective study	U.S.	Until 12/2021	45	38 (84)	17 (38)	MFC	≥ 10^–5^
Diaz-Pallares et al. (2020)	Retrospective study	Canada	01/2012 to 08/2018	34	18 (53)	3 in CR (50)^a^	MFC	Not reported
Kastritis et al. (2021a)	Retrospective study	Greece	05/2016 to 12/2019	51	51 (100)	23 (45)	NGF-MFC	3.1 × 10^–6^ to 2 × 10^–6^
Li et al. (2022)	Retrospective study	China	2012 to 07/2019	25	25 (100)	17 (68)	MFC	5 × 10^–5^ to 10^–5^
Muchtar et al. (2020)	Retrospective study	U.S	02/2012 to 11/2015	82	54 (66)	24 in VGPR/CR (44)	MFC	1 × 10^–4^ to 2 × 10^–5^
Palladini et al. (2021)	Retrospective study	Italy	04/2016 to 07/2019	92	92 (100)	42 (46)	NGF-MFC	≥ 10^–5^
Sarosiek et al. (2021)	Prospective study (NCT02716103)	U.S.	2016 to 2019	13	12 (92)	1 (8)	NGS	≥ 10^–6^
Sidana et al. (2020)	Retrospective study	U.S.	08/2017 to 11/2018	44	42 (95)	28 (64)	NGF-MFC	≥ 10^–5^
Staron et al. (2020)	Retrospective study	U.S.	02/2019 to 11/2019	65	65 (100)	29 (45)	MFC	≥ 10^–5^

*MFC* multiparameter flow cytometry, *NGF* next generation flow, *NGS* next generation sequencing, *CR* complete response, *VGPR* very good partial response

^a^9 patients achieved CR at MRD assessment in Diaz-Pallares et al. (2020), in which 3 were MRD negative, 3 were MRD positive, and 3 were MRD unknown

**Table 2:** Outcomes of the included studies

References	Cardiac response at MRD assessment (MRD^+^ vs. MRD^−^)	Renal response at MRD assessment (MRD^+^ vs. MRD^−^)	Disease progression after MRD assessment (MRD^+^ vs. MRD^−^)	Median follow-up time	PFS/OS (MRD^+^ vs. MRD^−^)
Chakraborty et al. (2022)	64% versus 92%	50% versus 60%	Not reported	22.5 months	55 months versus not reached in mMOD-PFS (*p* = 0.28)
Diaz-Pallares et al. (2020)	Not reported	Not reported	Not reported	24 months	No difference in PFS and OS among CR patients (*p* > 0.05)
Kastritis et al. (2021a)	73% versus 100%	87.5% versus 88%	Organ: 21% versus 4%; hematologic: 21% versus 0% during the follow-up. Progression rates at 1- and 2-year were acquired from the relevant Figures	24 months	68% versus 96% in PFS (*p* = 0.026)
Li et al. (2022)	25% versus 93%	50% versus 82%	Not reported	25.1 months	24.52 months versus 76.39 months in PFS (*p* = 0.004); no difference in OS (*p* = 0.2)
Muchtar et al. (2020)	83% versus 100% (in VGPR/CR patients)	68% versus 100% (in VGPR/CR patients)	Not reported	4.6 years	28% versus 88% in 3-year PFS (*p* < 0.001); 84% versus 96% in 3-year OS (*p* = 0.17)
Palladini et al. (2021)	75% versus 95%	62% versus 90%	Hematologic: 26% versus 2.4% in the first two years	23 months	Not reported
Sarosiek et al. (2021)	60% in MRD^+^	78% in MRD^+^	Not reported	Not reported	Not reported
Sidana et al. (2020)	22% versus 67%	89% versus 69%	Hematologic + organ: 36% versus 0% in the first year	14 months	64% versus 100% in 1-year PFS (*p* = 0.006)
Staron et al. (2020)	59% versus 75%	64% versus 88%	Not reported	Not reported	Not reported

*PFS* progression-free survival, *OS* overall survival, *mMOD-PFS* modified version of major organ deterioration-progression free survival

### Assessment of article quality

The methodological quality of the included studies was summarized in the Supplementary Table 1. Among the 9 included studies, the total score ranged from 14 to 19. The included studies were eligible for meta-analysis.

### Correlation of MRD status with the organ response rate in VGPR or CR patients

**Fig. 1:**
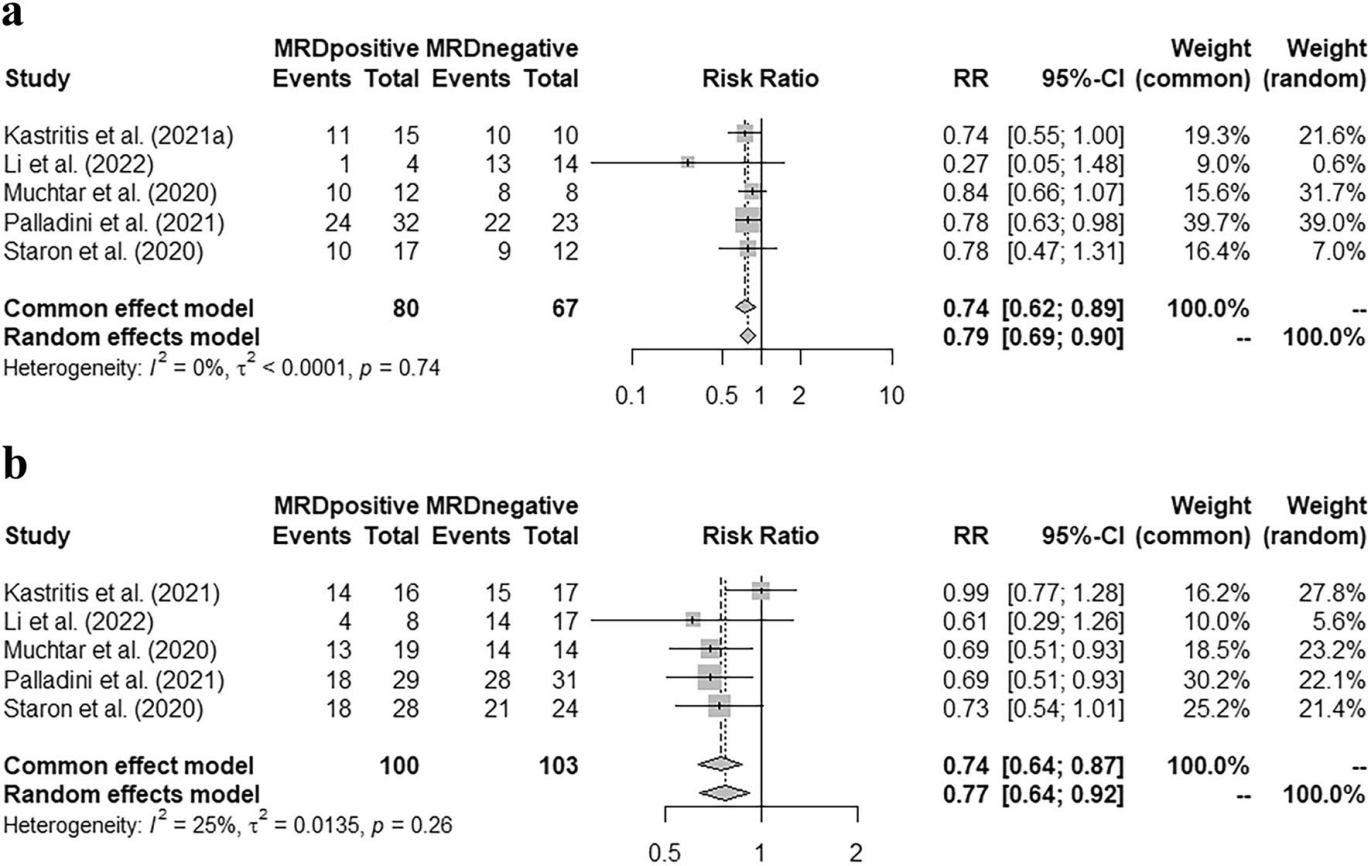
Correlation of MRD status with the organ response rate after achieving VGPR or CR. **a** Correlation of MRD status with cardiac response rate. **b** Correlation of MRD status with renal response rate

Five studies with 80 MRD-positive and 67 MRD-negative patients (all achieved VGPR or CR at MRD assessment), reported the cardiac response rate when performing the MRD detection, and MRD negativity was correlated with a higher cardiac response rate [Fig. 1a, pooled RR = 0.74 (95% CI 0.62–0.89)]. Meanwhile, these studies also reported the renal response rate in CR or VGPR patients who had renal involvement at baseline, and the pooled RR indicated a higher renal response rate with MRD negativity [Fig. 1b, pooled RR = 0.74 (95% CI 0.64–0.87)].

### Correlation of MRD status with disease progression

**Fig. 2:**
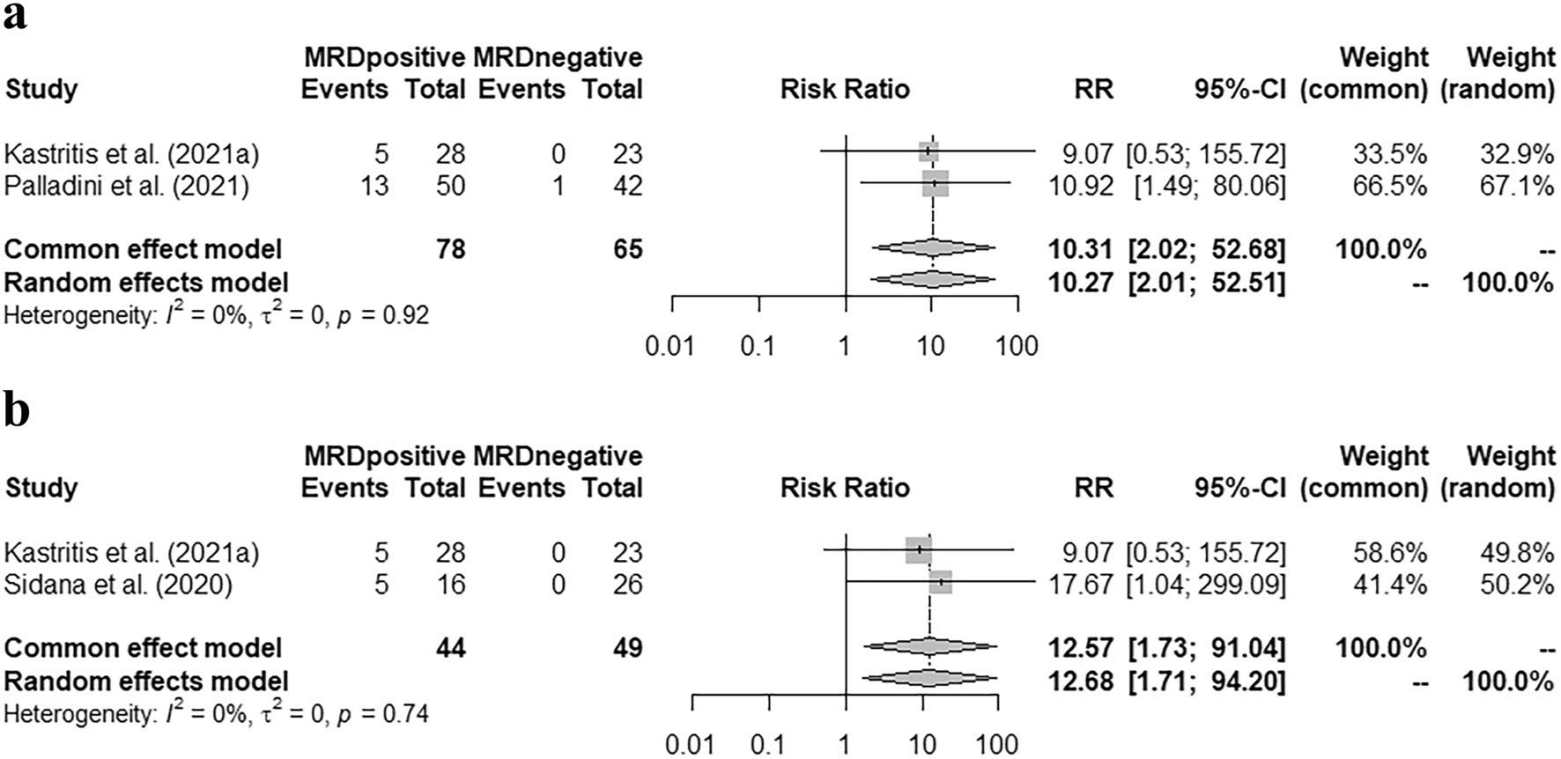
Correlation of MRD status with disease progression. **a** Pooled risk ratio of hematologic progression in the first two years grouped by MRD status. **b**: Pooled risk ratio of hematologic + organ progression in the first year grouped by MRD status

Kastritis et al. (2021a) and Palladini et al. (2021) demonstrated the hematologic progression rate in the first two years after the detection of MRD. The pooled RR indicated that patients with MRD positivity had a much higher risk of hematologic progression [Fig. 2a, pooled RR = 10.31 (95% CI 2.02–52.68)]. Similarly, a higher hematologic + organ progression rate in the first year was observed according to the studies by Kastritis et al. (2021a) and Sidana et al. (2020) [Fig. 2b, pooled RR = 12.57 (95% CI 1.73–91.04)].

### Correlation of MRD status with PFS and OS

**Fig. 3:**
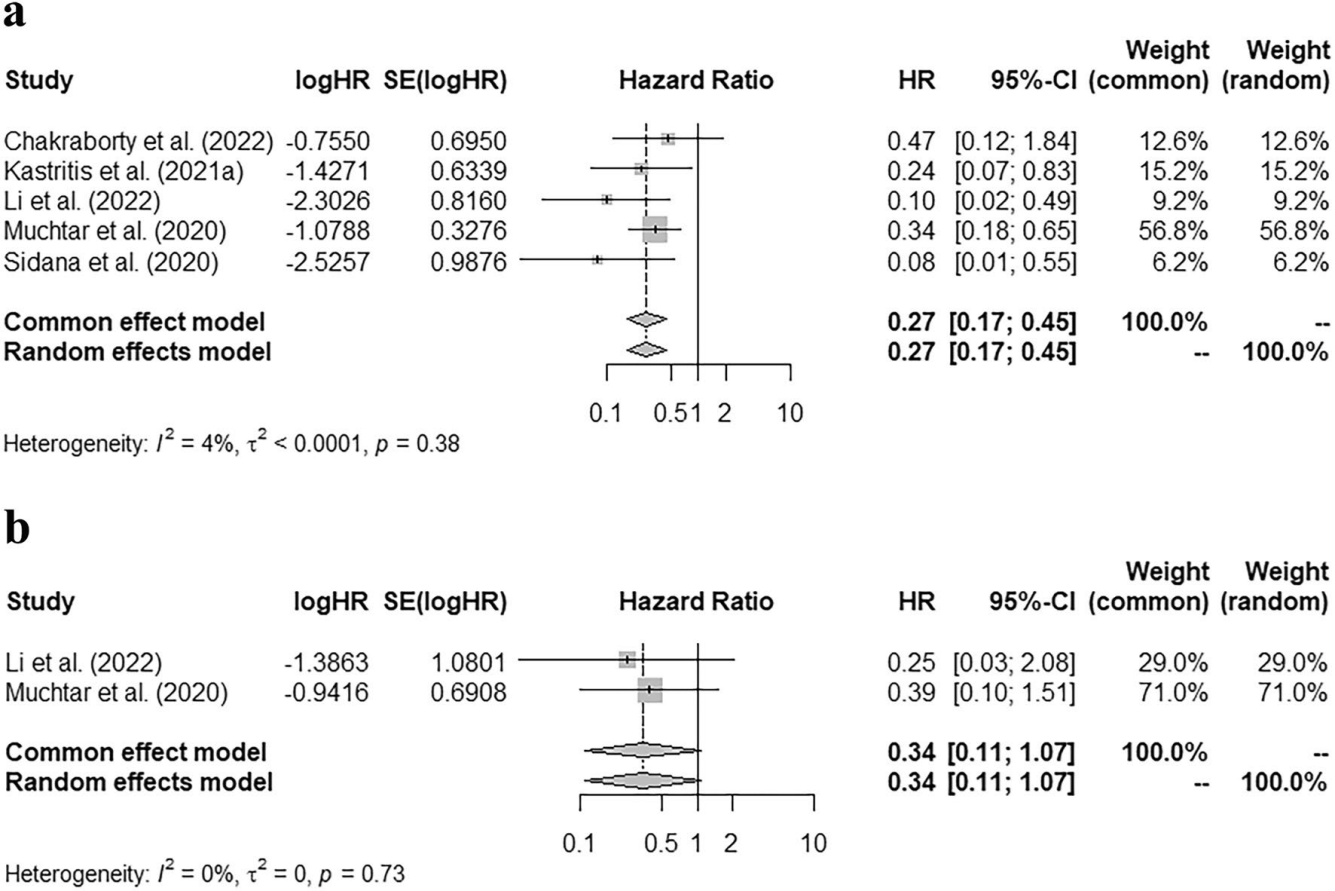
Correlation of MRD status with PFS and OS. **a** Pooled hazard ratio of PFS grouped by MRD status. **b** Pooled hazard ratio of OS grouped by MRD status

Five studies reported the p-value of PFS curve, the number of patients with progressive events, and the total number of MRD negative or positive patients. An approximate HR was calculated based on the data above. The pooled HR of PFS was 0.27 (95% CI 0.17–0.45) (Fig. 3a), which indicated that MRD negativity after treatment was correlated with a lower risk of disease progression during follow-up. The pooled HR of OS curve was calculated in a similar way. However, there was not a significant correlation between a better OS and MRD negativity [Fig. 3b, pooled HR = 0.34 (95% CI 0.11–1.07)].

### Publication bias analysis

Since AL amyloidosis is a rare disease, and the number of included studies was less than 10, we conducted an Egger's regression test to evaluate the potential publication bias. The *p* value of Egger's regression test was 0.354 (Supplementary Fig. S2), indicating that no potential publication bias was present in our study.

## Discussion

AL amyloidosis is a malignant disease characterized by abnormal plasma cell clone and amyloidogenic light chain deposition, causing hematologic and organ dysfunction. In recent years, MRD detection has been used to monitor patients with AL amyloidosis in the follow-up, and the value of MRD monitoring needs to be further analyzed and validated.

Our meta-analysis supported that once the patients achieved VGPR or CR, the recommended early therapy target, there was a statistical correlation between MRD status and the organ response rate, both in heart and kidney [pooled RR = 0.74 (95% CI 0.62–0.89), 0.74 (95% CI 0.64–0.87), respectively]. Previous studies have pointed out that, although some patients with AL amyloidosis achieved hematologic response after appropriate treatment, the recovery of organ function was not permitted and it might be due to the low level of clonal plasma cell burden (Staron et al. 2020; Szalat et al. 2021). Our study further supported that, a small burden of clonal plasma cells, with their continued production of monoclonal light chains, could still cause sustained organ damage and affect the patient's life quality. Therefore, our study highlighted the importance of reducing clonal plasma cell burden in the initial treatment.

In the studies we reviewed, Chakraborty et al. (2022) and Sidana et al. (2020) reported the organ response rate in negative MRD patients during the follow-up after MRD detection (May 2021 to 12/31/2021; 1 year after MRD detection, respectively). In the study of Chakraborty et al. (2022), the total cardiac or renal response rate in negative MRD patients did not improve during the follow-up after MRD detection; while the cardiac and renal ≥ VGPR rates increased by 16.7% and 4.4%, respectively. In addition, the total renal response rate in MRD negative patients increased by12% in Sidana et al. (2020). However, none of the two articles reported 100% organ response rate after MRD detection, which might be due to the short time-span of follow-up, or the irreversible organ damage caused by amyloid deposition. Therefore, more clinical studies with different time-span of follow-up are needed to evaluate the impact of MRD status on organ response after MRD detection, which may further validate the value of MRD status on therapeutic decision making.

The MRD status was also associated with the disease progression in the early follow-up. Our meta-analysis validated that patients with MRD-positivity had a higher risk of hematologic + organ progression in the first year after MRD detection [pooled RR = 12.57 (95% CI 1.73–91.04)]. Like other malignant diseases, the residual tumor cells can be the source of relapse and disease progression (Bertamini et al. 2021; Jongen-Lavrencic et al. 2018). Therefore, eliminating the residual clonal plasma cells as much as possible during induction and maintenance treatment, could reduce the risk of recurrence or progression.

In other hematological malignancies, such as multiple myeloma, the MRD status had been confirmed as a significant predictor in survival analysis, both in PFS and OS (Munshi et al. 2020). Our study supported the role of MRD negativity in predicting longer PFS [pooled HR = 0.27 (95% CI 0.17–0.45)]. As mentioned above, MRD-negative patients had a higher rate of organ response at the time of detection, and a lower risk of disease progression in the following 1 or 2 years; as a result, fewer patients deteriorated into organ malfunction in the early stage among the group with MRD negativity. Besides, smaller tumor burden was speculated in MRD-negative patients, according to those findings. Therefore, longer PFS was reasonable in this group of patients.

Although the MRD-negative group had a longer OS than the other, the difference was not statistically significant [pooled HR = 0.34 (95% CI 0.11–1.07)]. Our meta-analysis included the studies of Diaz-Pallares et al. (2020), Li et al. (2022) and Muchtar et al. (2020), which analyzed the impact of MRD status on the OS, but neither of them found a significant difference in the OS between the MRD-positive and -negative groups. The non-significance in OS may be due to the time span of follow-up. With the advancement of treatment regimens and the renewal of treatment methods, the median survival of AL amyloidosis has increased steadily in the past 40 years. The median survival of AL amyloidosis in 1980–1989, 1990–1999, 2000–2009, and 2010–2019 were 1.4, 2.6, 3.3, and 4.6 years, respectively (*p* < 0.001) (Staron et al. 2021). The use of autologous stem cell transplantation and proteasome inhibitors has improved the overall survival of AL amyloidosis (Huang et al. 2014; Kastritis et al. 2020). Besides, anti-CD 38 monoclonal antibody, such as Daratumumab, could further deepen the hematologic response and prolong survival free from major organ deterioration or hematologic progression (Kastritis et al. 2021b). Therefore, the difference in OS between various MRD statuses could get more significant when a longer follow-up span was set. Furthermore, since MRD status was associated with the response of impaired organs, the difference might be more significant in the subgroup with more serious organ dysfunction.

Our meta-analysis confirmed that MRD status in AL amyloidosis was related to organ response rate and the risk of disease relapse or progression. However, the depth of MRD detection may also affect the results of MRD status, thereby interfering with the accuracy of clinical decision-making. Multiparameter flow cytometry (MFC) is one of the most commonly used methods for MRD detection in AL amyloidosis. Quantification of bone marrow plasma cells using MFC in newly diagnosed AL amyloidosis could help predict patients’ prognosis (Paiva et al. 2011). The studies included in this meta-analysis used MFC for MRD detection with detection depths ranging from 2 × 10^–6^ to 1 × 10^–4^. In another study using matrix-assisted laser desorption/ionization-time-of-flight (TOF) mass spectrometry (MS) for detection of residual disease in AL amyloidosis, researchers found that even in patients who had achieved hematologic complete response and were negative for bone marrow flow cytometry, evidence of residual disease could still be found in 12% of the samples of the included patients; meanwhile, patients with positive residual disease had a higher risk of disease progression (at 50 months 75% vs. 13%, *p* = 0.003) (Dispenzieri et al. 2020). Therefore, a more suitable method or depth of MRD detection could guide the prediction of prognosis more accurately, and it is still an area that needs further research and exploration.

In recent years, the depth and accuracy of MRD detection have improved steadily with the advances in MRD technology. Our meta-analysis confirmed the clinical values of MRD detection in AL amyloidosis, and highlighted the importance of eliminating residual clonal plasma cells. However, some of the studies selected in our meta-analysis were retrospective, and the time span of follow-up might not be sufficiently long, so our results still require further confirmation by large-sized randomized clinical trials. With more advanced therapies and monitoring methods, patients with AL amyloidosis would meet better prognoses in the future.

## Supplementary Information

Below is the link to the electronic supplementary material.

Supplementary file1 (PDF 338 KB)

## Data Availability

The data underlying this article are available upon reasonable request to the corresponding author.
