# Supplementary material for: Minimal residual disease in systemic light chain amyloidosis: a systematic review and meta-analysis
Source: J Cancer Res Clin Oncol. 2024 Apr 15;150(4):193. doi: 10.1007/s00432-024-05733-2 (PMC11018658; doi:10.1007/s00432-024-05733-2)
Supplement: Supplementary file 1 — Supplementary file1 (PDF 338 KB) [file 432_2024_5733_MOESM1_ESM.pdf]

# Supplementary Text S1

## Literature search:

Embase: ('al amyloidosis'/exp OR 'al amyloidosis' OR 'amyloid light chain amyloidosis' OR 'amyloidosis, primary' OR 'immunoglobulin light chain amyloidosis' OR 'immunoglobulin light-chain amyloidosis' OR 'primary al amyloidosis' OR 'primary amyloidosis') AND ('minimal residual disease'/exp OR 'disease, minimal residual' OR 'minimal residual disease' OR 'minimum residual disease' OR 'neoplasm, residual' OR 'measurable residual disease' OR 'residual disease' OR 'residual disease, minimal' OR 'residual minimal disease' OR 'residual neoplasm')

Pubmed and Cochrane: (al amyloidosis OR amyloid light chain amyloidosis OR amyloidosis, primary OR immunoglobulin light chain amyloidosis OR immunoglobulin light-chain amyloidosis OR primary al amyloidosis OR primary amyloidosis) AND (minimal residual disease OR disease, minimal residual OR measurable residual disease OR minimum residual disease OR neoplasm, residual OR residual disease OR residual disease, minimal OR residual minimal disease OR residual neoplasm)

# Supplementary Text S2

## Equation of calculating O-E:

$$O - E = \frac{\sqrt{(Total\ observed\ events \times Analysed\ research \times Analysed\ control)}}{(Analysed\ research + Analysed\ control)} \times (\text{z score for } p\ value \div 2)$$

## Supplementary Fig. S1

**The process of literature search**

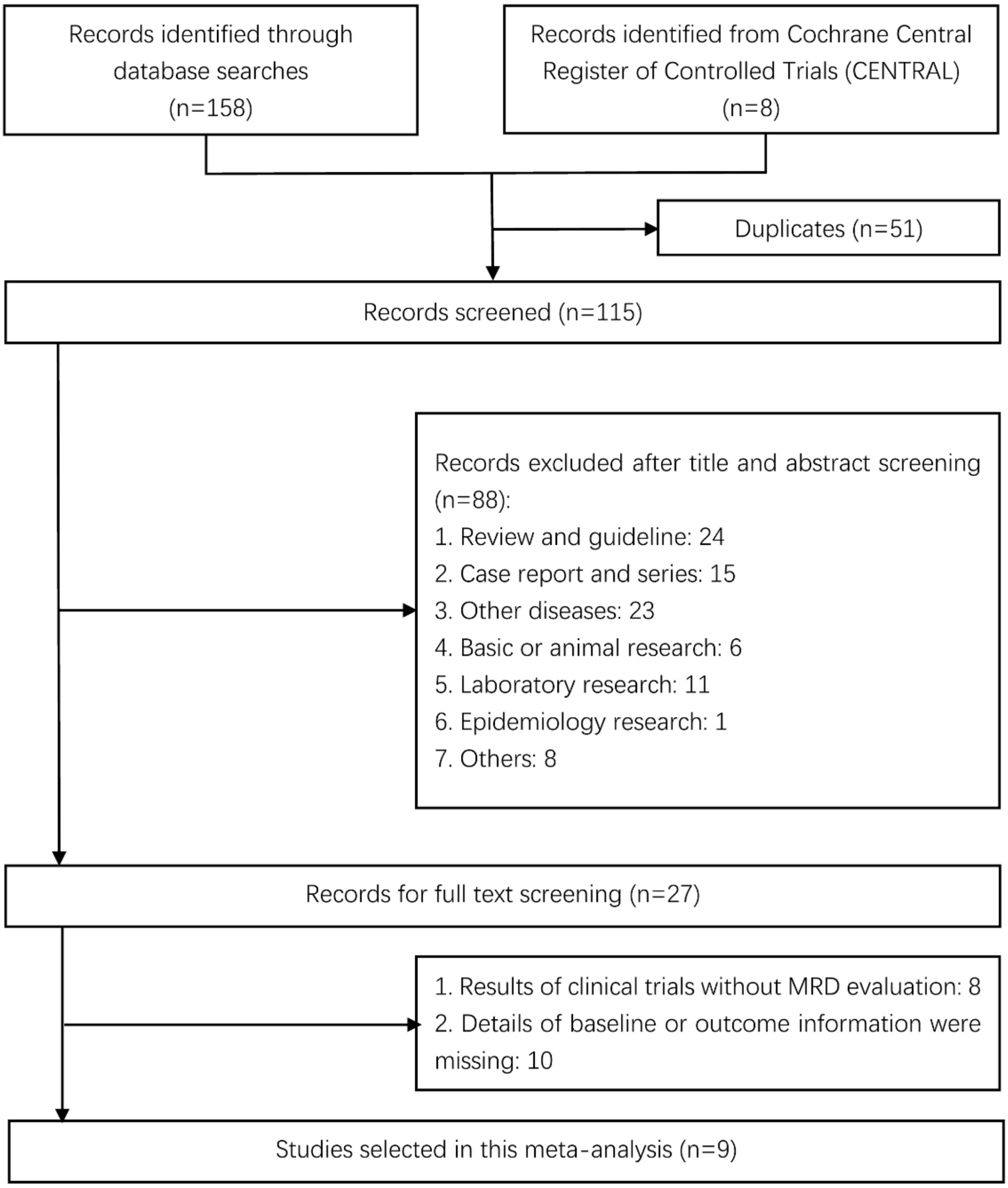

## Supplementary Fig. S2

### Egger's regression test of renal response rate in VGPR or CR patients

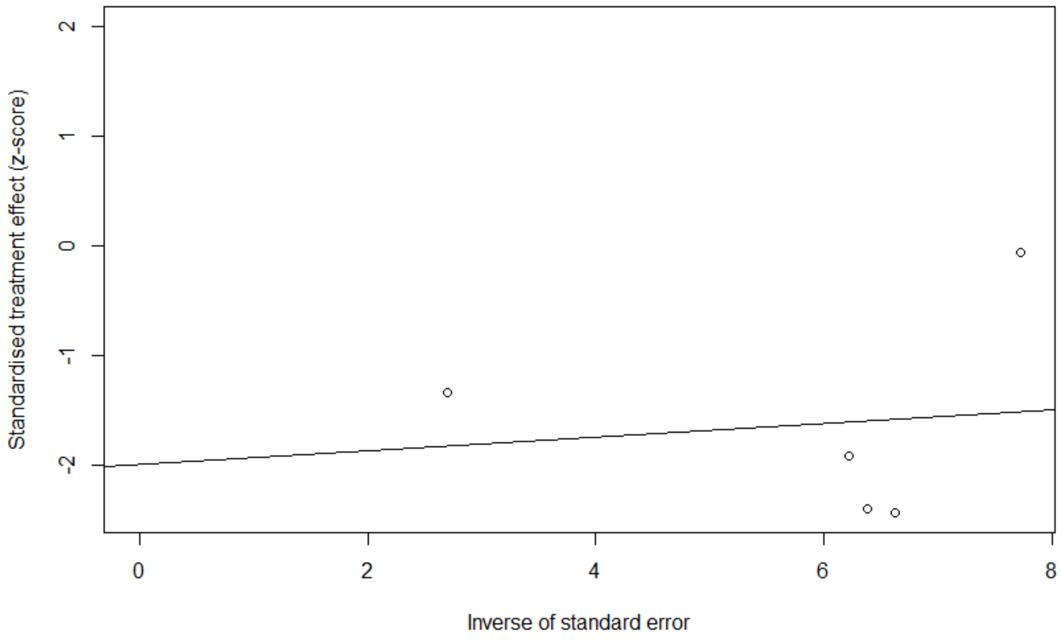


Test result: t = -1.09, df = 3, p-value = 0.3539

## Supplementary Table 1

**Total scores of each study assessed by MINORS**

| Study | 1 | 2 | 3 | 4 | 5 | 6 | 7 | 8 | 9 | 10 | 11 | 12 | Summary |
|---|---|---|---|---|---|---|---|---|---|---|---|---|---|
| Chakraborty et al. (2022) | 2 | 2 | 1 | 1 | 0 | 2 | 2 | 0 | 2 | 1 | 2 | 2 | 17 |
| Diaz-Pallares et al. (2020) | 2 | 2 | 2 | 2 | 0 | 2 | 2 | 0 | 2 | 1 | 0 | 2 | 17 |
| Kastritis et al. (2021a) | 2 | 2 | 2 | 2 | 0 | 1 | 2 | 0 | 2 | 1 | 1 | 2 | 17 |
| Li et al. (2022) | 2 | 2 | 2 | 2 | 0 | 2 | 2 | 0 | 2 | 2 | 1 | 2 | 19 |
| Muchtar et al. (2020) | 2 | 2 | 2 | 2 | 0 | 2 | 2 | 0 | 2 | 2 | 0 | 2 | 18 |
| Palladini et al. (2021) | 2 | 2 | 2 | 2 | 0 | 1 | 2 | 0 | 2 | 1 | 2 | 2 | 18 |
| Sarosiek et al. (2021) | 2 | 1 | 1 | 2 | 0 | 1 | 1 | 0 | 2 | 1 | 1 | 2 | 14 |
| Sidana et al. (2020) | 2 | 2 | 2 | 2 | 0 | 1 | 2 | 0 | 2 | 1 | 2 | 2 | 18 |
| Staron et al. (2020) | 2 | 2 | 2 | 2 | 0 | 2 | 2 | 0 | 2 | 1 | 2 | 2 | 19 |

MINORS: Methodological index for non-randomized studies

## Supplementary Table 2

### Baseline characteristics of the included patients

| Study | Average age | Male/Female | Mayo 2004, I/II/III | Mayo 2012, I/II/III/IV | Average NTproBNP (ng/L) | Average creatinine (μmol/L) | Average urine protein (g/day) | Cardiac/Renal involvement |
|---|---|---|---|---|---|---|---|---|
| Chakraborty et al. (2022) | 66 | Not reported | 14/10/21 | 17/8/12/8 | 1018 | 110 | Not reported | 26/24 |
| Diaz-Pallares et al. (2020) | 64 | 20/14 | Not reported | 5/8/7/12 | 1868.5 | 93 | 0.9 | 25/23 |
| Kastritis et al. (2021a) | 62 | 27/24 | 14/24/13 | Not reported | 1035 | Not reported | 0.39 | 25/33 |
| Li et al. (2022) | 59 | 15/10 | 10/7/8 | Not reported | 413 | 135 | 4.89 | 18/25 |
| Muchtar et al. (2020) | 61 | 56/26 | 37/32/13 | 34/27/6/15 | Not reported | Not reported | Not reported | 42/51 |
| Palladini et al. (2021) | 60 | 54/38 | Not reported | Not reported | Not reported | Not reported | Not reported | 59/61 |
| Sarosiek et al. (2021) | Not reported | Not reported | Not reported | Not reported | Not reported | Not reported | 0.08 | Not reported. |
| Sidana et al. (2020) | 63 | 26/18 | Not reported | Not reported | 926 | Not reported | 3.49 | 21/31 |
| Staron et al. (2020) | 61 | 41/24 | Not reported | Not reported | 140.0 (BNP) | 96 | 7.52 | 29/52 |

NTproBNP: N-terminal pro-brain natriuretic peptide.
